# Prosthetic replacement in femoral neck fracture in the elderly: Results and review of the literature

**DOI:** 10.4103/0019-5413.38583

**Published:** 2008

**Authors:** SKS Marya, R Thukral, Chandeep Singh

**Affiliations:** Max Institute of Orthopedics and Joint Replacement, Max Super Specialty Hospitals, 1, Press Enclave Road, Saket, New Delhi - 110 017, India

**Keywords:** Elderly patient, femoral neck fracture, hip arthroplasty

## Abstract

**Background:**

Intracapsular fractures of the proximal femur account for a major share of fractures in the elderly. The primary goal of treatment is to return the patient to his or her pre-fracture functional status. There are multiple internal fixation options (screws, dynamic hip screw plate or blade plates) and hemi and total hip arthroplasty. Open reduction and internal fixation has been shown to have a high rate of revision surgery due to nonunion and avascular necrosis. Hip replacement arthroplasty (hemi or total) is a viable treatment option.

**Materials and Methods:**

Eighty-four elderly patients (age >70 years) with a femoral neck fracture were treated over a five-year period (January 2001 to December 2006). Eighty of the 84 patients underwent some form of hip replacement after appropriate medical and anesthetic fitness.

**Results:**

We had good results in all the patients in terms of return to pre-fracture level of activity, independent ambulation and satisfaction with the procedure. Patients over the age of 80 years who underwent bipolar hemiarthroplasty all progressed well without any complication. Patients in their seventies underwent some form of total hip replacement and barring one case of deep infection, two cases of deep vein thrombosis and three cases of dislocation (which were managed conservatively), there were no real complications.

**Conclusion:**

Hip replacement (hemi or total) is a successful procedure for the elderly population over 70 years with femoral neck fractures. Return to pre-morbid level of activity and independent functions occur very swiftly, avoiding the hazards of prolonged incumbency. We have proposed a treatment algorithm following the results of treatment of this fracture in our series. We have also reviewed the different contemporary treatment options used (conservative treatment, cancellous screw fixation, Dynamic Hip Screw (DHS) fixation, hemi and total hip replacement) used for treatment of an elderly patient with of femoral neck fracture.

## INTRODUCTION

Intracapsular fractures of the proximal femur form a major share of fractures in the elderly.^1^ Osteoporosis, co-morbidities, increased incidence of trivial trauma increases the incidence and complicates the treatment of these fractures. This high incidence is due to weak bones and increased incidence of trivial trauma. People in this age group suffer from numerous illnesses that can aggravate the morbidity following fractures and complicate the treatment of these fractures. The treatment goal is to return the patient to his or her pre-morbid status of function. Increase in the average lifespan and improved medical facilities have greatly increased the incidence of these fractures.

Management of femoral neck fractures in elderly patients has been controversial. Femoral neck fractures have been considered ‘unsolvable fractures’ in the older era of orthopedics^2^ due to the high rate of associated complications, which include nonunion and avascular necrosis of the femoral head, among others. Presently, there are multiple surgical treatment options (cannulated screws, dynamic hip screw systems, blade plates, hemi and total hip arthroplasty) available. Intracapsular extent of the fracture, tenuous blood supply to the femoral head going through the neck and difficulty in maintaining fracture reduction have been cited as reasons for failure of fixation.^2^–^4^ Although treatment methods have been refined over the years, a consensus on the ideal treatment remains elusive.

Important factors to consider in choosing any treatment modality are intrinsic, viz. patient age, general medical condition, type of fracture; and extrinsic, viz. availability of facilities and socio-economic status.

Though non-operative treatment of these fractures has been documented,^1^ there are currently very few indications for the same (being limited to terminally ill patients or those who are bedridden and non-ambulatory). Surgical treatment has been established as the gold standard; however, the surgical option remains a dilemma. Open reduction and internal fixation has been shown to have a high rate of revision surgery due to nonunion and avascular necrosis.^2^–^6^ Hip replacement arthroplasty (partial or total) is emerging as the most viable treatment option.^7^–^11^

We present the results of treatment of this fracture in our series of 84 elderly patients over a five-year period. We have also reviewed the literature for different contemporary treatment options used (conservative treatment, cancellous screw fixation, Dynamic Hip Screw fixation, hemi and total hip replacement) in an elderly patient with femoral neck fracture. Based on the results of our study, we developed a treatment algorithm.

## MATERIALS AND METHODS

We have reviewed 84 elderly patients (age, >70 years) with a femoral neck fracture treated by a single surgeon over a five-year period (January 2001 to December 2006).

There were 49 male and 35 female patients of average age 78 years (age range, 72-90 years). Associated co-morbidities included combinations of diabetes mellitus, hypertension, chest disorders, cardiac disorders, renal diseases, hepatic diseases, neurological and psychiatric illnesses and one patient had had deep venous thrombosis in the past. Fourteen per cent (12 patients) had more than five coexisting morbidities and 65% (54 patients) had less than three co-morbid factors [Table 1].

**Table 1:** Number of patients having associated co-morbidities factors

Associated co-morbidities	Patients
Diabetes mellitus	52
Hypertension	66
Chest disorders	8
Cardiac disorders	24
Renal diseases	10
Hepatic disorders	2
Neurological disorders	10
Old DVT/PE	1

DVT - Deep vein thrombosis, PE - Pulmonary embolism

The patients had presented to us either immediately after a trivial fall or after being treated conservatively at home or outside over a period averaging two weeks (range, one day to six months). Four patients were either bedridden or wheelchair-bound or had severe co-morbidities and were treated conservatively with bed-rest and physiotherapy as tolerated. The remaining 80 patients underwent surgery [Table 2]. All patients underwent some form of hip replacement after appropriate medical and anesthetic clearance by a single surgeon.

**Table 2:** Implant used in our patients of different age groups

Age group	Implanted hip	Patients
>85 years (n =14)	Cemented bipolar	8
	Cementless bipolar (all high risk of infection)	3
	No surgery	3
81-85 years (n = 25)	Cemented total hip replacement	11
	Cemented femur, uncemented acetabulum (good bone quality, no cost constraint)	7
	Cemented bipolar	5
	Cementless bipolar	1
	No surgery	1
70-80 years (n = 45)	Cemented total hip replacement	32
	Cementless total hip (good bone quality, no cost constraint)	2
	Uncemented femur, uncemented acetabulum (good bone quality, desirous of greater mobility, physiologically more active, no cost constraint)	4
	Cemented bipolar	6
	Cementless bipolar (old DVT 1 history, high risk for infection)	1

We opted for total hip arthroplasty procedure in the majority of our patient population (where life expectancy was greater than five years), all those with pre-existing acetabular pathology (inflammatory or arthritic). All patients were offered the options of cemented, hybrid and cementless fixation, as well as alternative implant tribological characteristics (metal-on-metal, anatomic hip replacement, etc). Final decision rested on factors ranging from patient choice of range of motion, capabilities expected post-surgery, economics, bone density and implant availability. In general, patients with better bone stock and lesser number of co-morbid factors were offered cemented or hybrid hips; whereas those with poor bone stock and greater number of co-morbid factors were treated with cementless hips.

**Figure 1:**
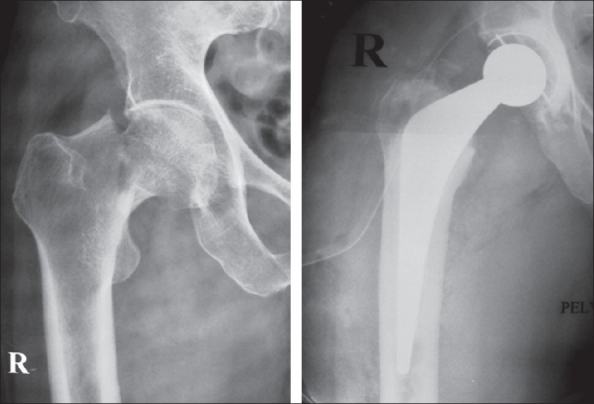
Preoperative and postoperative cemented total hip arthroplasty for fracture neck femur

**Figure 2:**
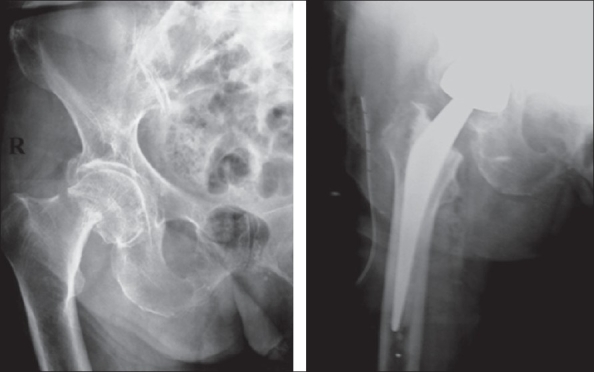
Preoperative and postoperative cemented bipolar arthroplasty for fracture neck femur

**Figure 3:**
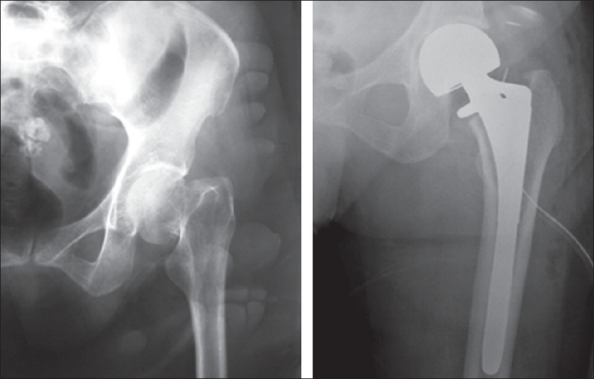
Preoperative and postoperative cementless bipolar arthroplasty for fracture neck femur

**Figure 4:**
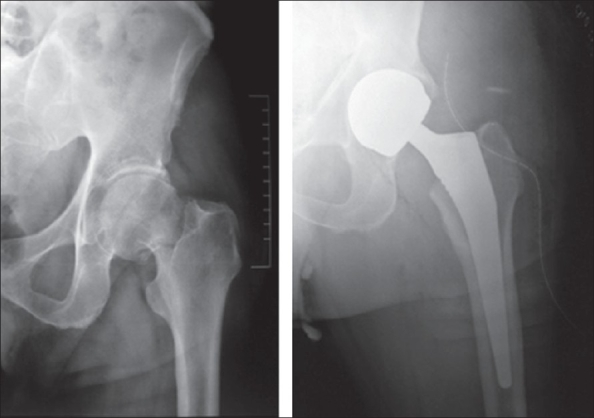
Preoperative and postoperative cementless total hip arthroplasty for fracture neck femur

**Chart 1:**
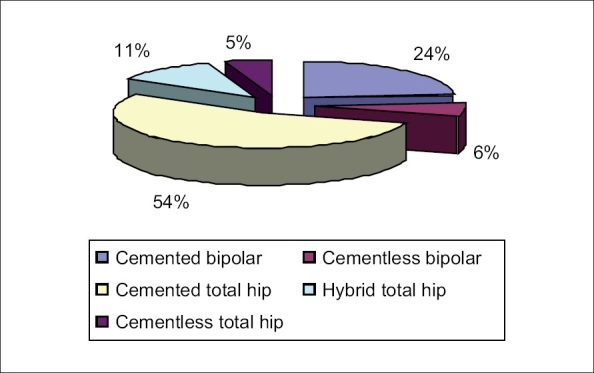
Pie chart depicting percentage of specific implant usage in the operated group

Cemented implants accounted for nearly 78% of all operated cases. The implants used included the cemented total hip (C-stem with Ogee cup, DePuy, Johnson and Johnson) in 54% (43 patients) [Figure 1], the cemented bipolar system (DePuy, Johnson and Johnson) in 24% (19 patients) [Figure 2], cementless system (Corail stem, DePuy, Johnson and Johnson) in 11% (nine patients, bipolar in five and total hip in four patients) [Figures 3–4]. The cementless system was used in patients who had either high risk of infection (due to past or recent history) or previous history of deep vein thrombism (DVT) or who had no cost constraints and were desirous of excessive mobility [Table 3 and Chart 1]. Nine patients underwent hybrid hip replacement (cemented femur and uncemented acetabular cup) (DePuy C-stem with Duraloc cup, Johnson and Johnson).

**Table 3:** Complications in our patient series

Complications	Patients
Deep infection	1
Superficial infection	5
Dislocation	3
Thigh pain	2
Bedsore formation	5
DVT	2
Acute confusional state	7
Urinary retention	8
Chest infection	6
Constipation	10

DVT - Deep vein thrombosis

Postoperative protocol for patients with cemented implants (bipolar or total hips) involved full weight-bearing as soon as possible (as per patient ability to stand supported) and active hip and knee exercises. Patients with hybrid hip replacements were initially mobilized to partial weight-bearing for three weeks and then graduated to full weight-bearing over the next three weeks. Patients with cementless implants (bipolar or total hips) were mobilized to non-weight-bearing for three weeks, partial weight-bearing for the following nine weeks and then allowed full weight-bearing without support. Active hip abduction exercises were initiated at six weeks.

Patients were reviewed at two weeks (for staple removal), six weeks, three months, six months, 12 months, 24 months and 60 months and assessed using clinical and radiological criteria. Clinical criteria used were absence of pain and limp, as well as the ability to perform activities of daily living independently and the Harris Hip scores (performed to quantify results only). All patients were studied radiologically for signs of loosening, subsidence (cemented implants), lysis and osteointegration (cementless implants).

## RESULTS

We had good results in the patients that we treated, in terms of return to pre-fracture level of activity, independent ambulation and satisfaction with the procedure. We used the anterolateral approach in all patients and the blood loss averaged 184 ml (range, 100-500 ml). Harris Hip scores of our 80 operated patients (documentation purposes only) averaged 81 (range, 70-94) at final follow-up.

The outline of our treatment algorithm had been initially prepared following conclusions derived from literature review and after analyzing the results of our study, has been modified to the protocol described in our paper.

Hemiarthroplasty was reserved for patients of age more than 80 who were independently mobile, had severe co-morbidities and needed excessive movements. We performed cemented bipolar hemi-arthroplasties in 19 patients and cementless bipolar hip replacement in five. These included those with previous deep vein thrombosis (DVT) or high risk of Pulmonary Emobolism, evidence of recent or past infection in the hip. This did not in any way delay the mobilization protocol. No patient underwent cementless Austin-Moore or Thompson prosthesis implantation. All patients proceeded well without any complication.

The complications that we encountered included one case of deep infection necessitating one episode of debridement and six weeks of parenteral antibiotics, two cases of DVT (managed with heparinization and delayed rehabilitation) but no fatal PE, three dislocations (all managed by closed reduction and post-reduction hip abduction bracing for six to eight weeks), two instances of thigh pain (all in the cementless subgroup), five patients developed superficial bedsores (which healed without sequelae) and seven patients developed acute confusional states (dyselectrolytemia, encephalopathy) [Table 3].

Although the follow-up period is short (average, five years, range one to six and a half years) and our study is non-randomized (limiting the efficacy of our results), non-controlled and retrospective, the results were consistent with contemporary literature.^8^,^11^–^15^ We have, on the basis of these results and review of the literature, devised a treatment algorithm for the management of this very common fracture of the elderly at our institution [Flowchart 1]. However, a prospective randomized controlled study is needed to test the usefulness of the algorithm.

**Flowchart 1:**
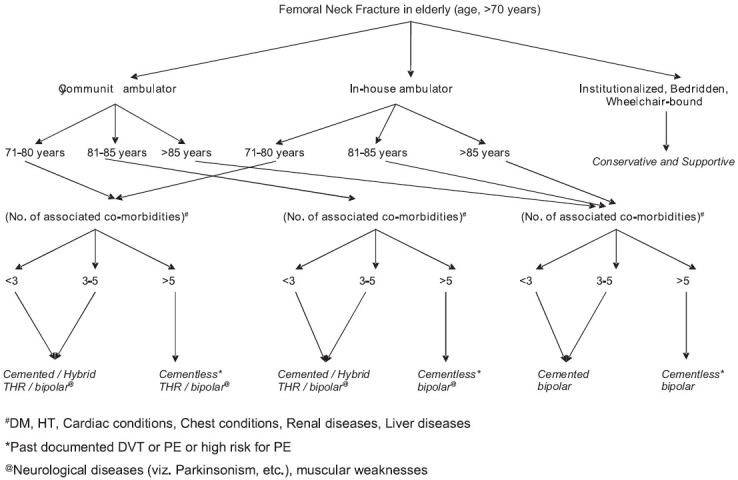
Treatment algorithm for hip replacement in displaced femoral neck fracture in the elderly

## DISCUSSION

Hip fractures in the elderly patient group result in implications in medicine, rehabilitation, psychiatry and healthcare economics. Conservative treatment is fraught with all the complications of prolonged recumbency viz. chest infections, formation of pressure sores and disuse osteoporosis. Non-operative management may be preferable for non-ambulatory, institutionalized patients with marked dementia who experience minimal discomfort within the first few days after the injury.^1^ Such patients’ “return to pre-injury level of function” is better accomplished without surgery. However, early mobilization is essential to avoid the associated complications. The number of patients falling into this category is usually quite small.^1^

Amongst the surgically treated group, the methods preferred are either internal fixation or various forms of arthroplasty (hemi or total). Most recent randomized studies have demonstrated high re-operation rates (34-43%) following reduction and fixation of displaced intracapsular hip fractures.^7^–^8^,^15^,^16^ The most common reasons for the re-operations were fixation failure and nonunion. Although osteonecrosis is a well-recognized complication, it is not the most common cause of re-operations. The other randomized studies included patients with limited mobility or cognitive function and it is often assumed that healthy older patients have a lower complication rate following reduction and fixation. Poorer outcomes and higher costs following fixation indicate that it is not cost-effective compared with either bipolar hemiarthroplasty or total hip replacement, unless there is an increase in revision rates across the procedures. Parker *et al*.,^4^ in a review of displaced femoral neck fractures, stated that for those aged less than 50-60 years preservation of the femoral head is paramount. With increasing age the arguments against arthroplasty diminish since the life expectancy of the patient becomes less than that of the arthroplasty and the functional demands on the hip are less. The incidence of nonunion increases progressively with age, while symptomatic avascular necrosis is less common in the elderly.^2^–^3^ Johansson *et al*.,^17^ in a prospective randomized trial comparing internal fixation with arthroplasty produced conflicting results. They suggested that both methods of treatment produce comparable final outcomes. Internal fixation is associated with a marginally lower mortality but at the expense of an increased rate of re-admission and re-operation. Both approaches are acceptable and surgeons must choose which method is best in their hands. Specific co-morbidity may tip the balance in favor of internal fixation. The presence of chronic sepsis, such as leg ulcers or an indwelling urinary catheter, is not an absolute contraindication to arthroplasty, but may lead the clinician to favor internal fixation.

Randomized trials of patients with limited mobility or impaired cognitive function have suggested that a unipolar cementless hemiarthroplasty (like the Austin-Moore or Thompson prosthesis) is the treatment of choice.^7^–^11^ The best choice for orthopedic management of patients who are 60 years of age or older and are otherwise healthy is still somewhat controversial.^4^ Reduction and fixation, bipolar hemiarthroplasty (with or without cement) and total hip replacement (cemented, hybrid or cementless) are the usual alternatives under consideration. The use of these options varies considerably among different surgeons and centers. Each has advantages and drawbacks. There is considerable evidence demonstrating better functional outcome and less need for re-operation with hip arthroplasty compared to internal fixation in the treatment of displaced femoral neck fractures in the elderly.^13^,^18^

It is clear that a patient with antecedent symptomatic osteoarthritis or inflammatory arthritis of his/her hip, who subsequently suffers a displaced subcapital hip fracture, would benefit most from a total hip replacement compared to a hemiarthroplasty.^19^ In addition, a patient who suffers a pathological femoral neck fracture with concomitant acetabular pathology should have a total hip replacement performed. When choosing between total hip and hemiarthroplasty it has to be borne in mind that total hip arthroplasty gives a better functional outcome in the active, independent senior citizen, but has a higher rate of dislocation.^19^ Hemiarthroplasty results in fewer dislocations, a shorter operating time and less need for blood transfusions, but there is a risk that acetabular erosion will limit the pain-free life of the implant.^19^,^20^ A bipolar hemiarthroplasty has the potential advantage of reducing the risk of acetabular wear for patients with a life expectancy of more than five years. For those aged over 80 years or who are inactive, the bipolar joint is probably of some benefit.^21^ Its disadvantages are that it is more expensive and although the rate of dislocation is similar to that for a unipolar hemiarthroplasty, closed reduction may not be possible in the event of a dislocation episode.^4^

The potential advantage of using total hip replacement relates to its highly predictable results, with survivorship of greater than 90% at 10 years and its unparalleled results in terms of pain relief and overall function. In addition, the use of total hip replacement avoids the potential need for revision secondary to acetabular pain from ongoing acetabular erosion.^13^,^17^ The potential disadvantages include the increased cost, increased surgical time and blood loss (which may lead to increased morbidity or mortality) and the potential increased rate of dislocation compared to hemiarthroplasty. Several studies from Scandinavia,^8^,^12^,^14^,^17^ where total hip replacement is commonly used to treat hip fractures, reporting on series of patients with displaced subcapital hip fractures randomized to receive either internal fixation or some form of arthroplasty (hemi or total), have concluded that the overall re-operation rate was significantly less and general function was considerably better in those patients receiving a total hip arthroplasty. The rate of dislocation ranged from 2 to 22% (comparable to the dislocation rate of 5.3% in our total hip series) and was linked to both the surgical approach and the mental status of the patient.^12^,^14^,^17^

What remains unclear is whether certain hip fracture patients, with no preexisting hip pathology, would benefit from total rather than hemiarthroplasty. Rogmark *et al*.^8^ have suggested that patients between the ages of 70-80 years, who live in their own home, who do not require any walking aids and are alert mentally represent the ideal candidates for total hip replacement. In contrast, those patients who are greater than 80 years of age, who live in a nursing home, who require ambulatory assistance and are mentally confused are best treated with hemihip replacement. This concept seems logically true, but needs further prospective randomized controlled trials to be regarded as dictum.

An argument against primary arthroplasty has been the possibility of increased postoperative mortality. Holmberg *et al*.^22^ found a higher mortality three weeks after hemiarthroplasty than after internal fixation, but their groups were not comparable since the mean age of the patients in the hemiarthroplasty group was six years older. The meta-analysis by Lu-Yao *et al*.^23^ did not find a significant difference, but did note a slightly higher mortality 30 days after hemiarthroplasty compared with internal fixation. Whereas, Hudson *et al*.^24^ found a higher mortality after internal fixation than after hemiarthroplasty when adjusted for age, gender and co-morbidities, but the selection criteria for the treatment in that study were not described. Often, the old and weak patient is given a hemiarthroplasty and the younger patient internal fixation and their mortality risks cannot be compared. Men have a higher mortality than women, as has been previously reported by Eiskjaer and Ostgard^25^ and Holmberg *et al*.^22^

According to a prospective randomized comparative study by Keating grant and Masson *et al*.,^15^ of the treatment of displaced femoral neck fractures in the elderly with internal fixation, hemi or total hip arthroplasty, the internal fixation procedure was found to be associated with a high rate of revision surgery and an inferior functional outcome compared with that of arthroplasty. This trend is particularly evident for younger patients (60-74 years old). Although the open reduction and internal fixation group had the lowest acute-admission costs (with less expensive implants, shorter operative time and shorter initial hospital stays), the greatly increased need for re-admissions and re-operations result in this management option having the highest costs overall. Differences between the two types of arthroplasty are less marked; however, the functional outcome at two years was significantly better following total hip replacement. The reasons cited for the apparent functional deterioration in the bipolar hemiarthroplasty group is early acetabular erosion associated with the bipolar head.

Similarly, other randomized studies^5^–^12^,^16^,^17^,^26^–^30^ describing the management of displaced intracapsular hip fractures have reported that the best clinical and functional outcomes have been observed after total hip replacement. This was not a popular method of treating these fractures in the past, because of a perception that it was associated with an unacceptably high rate of prosthetic dislocation. Recent meta-analysis, however, showed a mean rate of dislocation of 6.9%.^4^ Although higher than what is expected after arthroplasty for primary osteoarthritis, it is still acceptably low.

Our study, though non-randomized, non-controlled and retrospective, demonstrates acceptable results consistent with contemporary studies.^8^,^11^,^12^–^14^,^24^ In summary, hip replacement (hemi or total) is a successful procedure for the elderly population over 70 years with femoral neck fractures. Return to pre-morbid level of activity and independent functions occur very swiftly, avoiding the hazards of prolonged incumbency.

## References

[1] Holmberg S, Kalen R, Thorngren KG (1987). Treatment and outcome of femoral neck fractures: An analysis of 2418 patients admitted from their own homes. Clin Orthop Relat Res.

[2] Barnes R, Brown JT, Garden RS, Nicoll EA (1976). Subcapital fractures of the femur: A prospective review. J Bone Joint Surg Br.

[3] Parker MJ (1994). Prediction of fracture union after internal fixation of intracapsular femoral neck fractures. Injury.

[4] Parker MJ (2000). The management of intracapsular fractures of the proximal femur. J Bone Joint Surg Br.

[5] Skinner P, Riley D, Ellery J, Beaumont A, Coumine R, Shafighian B (1989). Displaced subcapital fractures of the femur: A prospective randomized comparison of internal fixation, hemiarthroplasty and total hip replacement. Injury.

[6] van Vugt AB, Oosterwijk WM, Goris RJ (1993). Osteosynthesis versus endoprostheses in the treatment of unstable intracapsular hip fractures in the elderly: A randomized clinical trial. Arch Orthop Trauma Surg.

[7] Davison JN, Calder SJ, Anderson GH, Ward G, Jagger C, Harper WM (2001). Treatment for displaced intracapsular fracture of the proximal femur: A prospective, randomized trial in patients aged 65 to 79 years. J Bone Joint Surg Br.

[8] Rogmark C, Carlsson Å, Johnell O, Sernbo I (2002). A prospective randomized trial of internal fixation versus arthroplasty for displaced fractures of the neck of the femur: Functional outcome for 450 patients at two years. J Bone Joint Surg Br.

[9] Søreide O, Mölster A, Raugstad TS (1979). Internal fixation versus primary prosthetic replacement in acute femoral neck fractures: A prospective, randomized clinical study. Br J Surg.

[10] Sikorski JM, Barrington R (1981). Internal fixation versus hemiarthroplasty for the displaced subcapital fracture of the femur: A prospective randomized study. J Bone Joint Surg Br.

[11] Parker MJ, Pryor GA (2000). Internal fixation or arthroplasty for displaced cervical hip fractures in the elderly: A randomized controlled trial of 208 patients. Acta Orthop Scand.

[12] Tidermark J, Ponzer S, Svensson O, Soderqvist A, Tornkvist H (2003). Internal fixation compared with total hip replacement for displaced femoral neck fractures in the elderly: A randomized, controlled trial. J Bone Joint Surg Br.

[13] Bhandari M, Devereaux PJ, Swiontkowski MF, Tornetta P, Obremskey W, Koval KJ (2003). Internal fixation compared with arthroplasty for displaced fractures of the femoral neck: A meta-analysis. J Bone Joint Surg Am.

[14] Ravikumar KJ, Marsh G (2000). Internal fixation versus hemiarthroplasty versus total hip arthroplasty for displaced subcapital fractures of femur: 13 year results of a prospective randomized study. Injury.

[15] Keating JF, Grant A, Masson M, Scott NW, Forbes JF (2006). Randomized comparision of reduction and fixation, bipolar hemiarthroplasty and total hip arthroplasty in the treatment of displaced intracapsular hip fractures in healthy older patients. J Bone Joint Surg Am.

[16] Parker MJ, Khan RJ, Crawford J, Pryor GA (2002). Hemiarthroplasty versus internal fixation for displaced intracapsular hip fractures in the elderly: A randomized trial of 455 patients. J Bone Joint Surg Br.

[17] Johansson T, Jacobsson SA, Ivarsson I, Knutsson A, Wahlstrom O (2000). Internal fixation versus total hip arthroplasty in the treatment of displaced femoral neck fractures: A prospective randomized study of 100 hips. Acta Orthop Scand.

[18] Roden M, Schon M, Fredin H (2003). Treatment of displaced femoral neck fractures: A randomized minimum 5-year follow-up study of screws and bipolar hemi prostheses in 100 patients. Acta Orthop Scand.

[19] Squires B, Bannister G (1999). Displaced intracapsular neck of femur fractures in mobile independent patients: Total hip replacement or hemiarthroplasty?. Injury.

[20] Dorr LD, Glousman R, Sew Hoy AL, Vanis R, Chandler R (1986). Treatment of femoral neck fractures with total hip replacement versus cemented and noncemented hemiarthroplasty. J Arthroplasty.

[21] Calder SJ, Anderson GH, Jagger C, Harper WM, Gregg PJ (1996). Unipolar or bipolar prosthesis for displaced intracapsular hip fracture in octogenarians: A randomized prospective study. J Bone Joint Surg Br.

[22] Holmberg S, Conradi P, Kalen R, Thorngren KG (1986). Mortality after cervical hip fracture: 3002 patients followed for 6 years. Acta Orthop Scand.

[23] Lu-Yao GL, Keller RB, Littenberg B, Wennberg JE (1994). Outcomes after displaced fractures of the femoral neck: A meta-analysis of one hundred and six published reports. J Bone Joint Surg Am.

[24] Hudson JI, Kenzora JE, Hebel JR, Gardner JF, Scherlis L, Epstein RS (1998). Eight-year outcome associated with clinical options in the management of femoral neck fractures. Clin Orthop Relat Res.

[25] Eiskjaer S, Ostgard SE (1991). Risk factors influencing mortality after bipolar hemiarthroplasty in the treatment of fracture of the femoral neck. Clin Orthop Relat Res.

[26] Jensen J, Rasmussen T, Christensen S, Holm-Moller S, Lauritzen J (1984). Internal fixation or prosthetic replacement in fresh femoral neck fractures. Acta Orthop Scand.

[27] Jonsson B, Sernbo I, Carlsson A, Fredin H, Johnell O (1996). Social function after cervical hip fracture: A comparison of hook-pins and total hip replacement in 47 patients. Acta Orthop Scand.

[28] Neander G, Adolphson P, von Sivers K, Dahlborn M, Dalen N (1997). Bone and muscle mass after femoral neck fracture: A controlled quantitative computed tomography study of osteosynthesis versus primary total hip arthroplasty. Arch Orthop Trauma Surg.

[29] Van Dortmont LM, Douw CM, van Breukelen AM, Laurens DR, Mulder PG, Wereldsma JC (2000). Cannulated screws versus hemiarthroplasty for displaced intracapsular femoral neck fractures in demented patients. Ann Chir Gynaecol.

[30] Puolakka TJ, Laine HJ, Tarvainen T, Aho H (2001). Thompson hemiarthroplasty is superior to Ullevaal screws in treating displaced femoral neck fractures in patients over 75 years: A prospective randomized study with two-year follow-up. Ann Chir Gynaecol.

